# Norepinephrine Drives Persistent Activity in Prefrontal Cortex via Synergistic α1 and α2 Adrenoceptors

**DOI:** 10.1371/journal.pone.0066122

**Published:** 2013-06-13

**Authors:** Zizhen Zhang, Steven Cordeiro Matos, Sonia Jego, Antoine Adamantidis, Philippe Séguéla

**Affiliations:** 1 Montreal Neurological Institute, Alan Edwards Centre for Research on Pain, Department of Neurology and Neurosurgery, McGill University, Montreal, Quebec, Canada; 2 Douglas Mental Health University Institute, Department of Psychiatry, McGill University, Montreal, Quebec, Canada; Vanderbilt University Medical Center, United States of America

## Abstract

Optimal norepinephrine levels in the prefrontal cortex (PFC) increase delay-related firing and enhance working memory, whereas stress-related or pathologically high levels of norepinephrine are believed to inhibit working memory via α1 adrenoceptors. However, it has been shown that activation of Gq-coupled and phospholipase C-linked receptors can induce persistent firing, a cellular correlate of working memory, in cortical pyramidal neurons. Therefore, despite its importance in stress and cognition, the exact role of norepinephrine in modulating PFC activity remains elusive. Using electrophysiology and optogenetics, we report here that norepinephrine induces persistent firing in pyramidal neurons of the PFC independent of recurrent fast synaptic excitation. This persistent excitatory effect involves presynaptic α1 adrenoceptors facilitating glutamate release and subsequent activation of postsynaptic mGluR5 receptors, and is enhanced by postsynaptic α2 adrenoceptors inhibiting HCN channel activity. Activation of α2 adrenoceptors or inhibition of HCN channels also enhances cholinergic persistent responses in pyramidal neurons, providing a mechanism of crosstalk between noradrenergic and cholinergic inputs. The present study describes a novel cellular basis for the noradrenergic control of cortical information processing and supports a synergistic combination of intrinsic and network mechanisms for the expression of mnemonic properties in pyramidal neurons.

## Introduction

The prefrontal cortex (PFC) plays a significant role in high order brain functions such as working memory [1]–[3], a process that refers to the ability to maintain relevant information in a temporary buffer to guide behaviour. Dysfunction of PFC neural networks occurs in many psychiatric disorders including attention deficit hyperactivity disorder and post-traumatic stress disorder (PTSD) which are associated with hypersensitivity to acute stress and deficits in working memory. It has long been known that working memory function is correlated with delay-related persistent neuronal firing and increased fMRI signal in the PFC of animals and human subjects performing working memory tasks [4]–[9]. Persistent firing which outlasts the original stimulus can be sustained via recurrent network excitation within microcircuits of layer 2/3 pyramidal neurons of the PFC or from afferent inputs of subcortical areas [3], [10], [11]. *In vivo* and *in vitro* experiments have shown that persistent firing can also be observed following a slow afterdepolarization in the presence of muscarinic or metabotropic glutamate receptor activation [12]–[19] Several reports have confirmed that pyramidal neurons exhibit intrinsic persistent firing independent of ionotropic synaptic transmission [20]–[24]. Despite the known impact of stress and imbalanced monoamine levels on PFC function, the mechanisms underlying the modulation of intrinsic persistent firing by norepinephrine (NE) have not been addressed.

The PFC receives dense noradrenergic innervation from the locus coeruleus. Optimal levels of NE released during waking have been shown to enhance neuronal activity in the PFC and improve spatial working memory performance in rats and monkeys via the activation of postsynaptic α2 adrenoceptors [1], [25], [26]. Activation of the phospholipase C pathway by postsynaptic metabotropic Gq-coupled receptors such as muscarinic receptors or mGluR5 induces long-lasting persistent neuronal firing in pyramidal neurons of the entorhinal cortex [20], [24] as well as the PFC [22], [27]. However, recent studies have reported that activation of postsynaptic Gq-coupled α1 adrenoceptors and PKC inhibits persistent activity of prefrontal pyramidal neurons and impairs working memory performance in monkeys [28], [29]. Therefore, in order to clarify the exact role of α1 adrenoceptors and to understand the mechanism of noradrenergic modulation on prefrontal functions, the present study investigated the effect of NE on pyramidal neurons at the single cell level using patch clamp recording combined with optogenetic activation of noradrenergic fibers in acute brain slices. We found that both exogenous NE and endogenous NE released from the locus coeruleus induce persistent firing in pyramidal neurons of the PFC. We provide evidence that noradrenergic persistent responses are mainly mediated by a synergy between presynaptic α1 adrenoceptor-mediated enhancement of glutamate release and postsynaptic α2 adrenoceptor-mediated inhibition of hyperpolarization-activated cyclic nucleotide-gated (HCN) cation channels.

## Materials and Methods

### Electrophysiological Recording

All experimental procedures were approved by the McGill University Animal Care Committee and were in compliance with the guidelines of the Canadian Council on Animal Care. Acute brain slices were obtained from adult Long-Evans rats (20–23 days old) (Charles River Canada, Saint-Constant, Quebec, Canada) or transgenic mice expressing ChR2 (see below) according to a procedure described previously [22]. Animals were anesthetized with a ketamine:xylazine cocktail (60∶5 mg/kg) and transcardially perfused with ice-cold choline chloride–based artificial cerebrospinal fluid (cutting solution) consisting of (in mM): 110 choline-Cl, 1.25 NaH_2_PO_4_, 25 NaHCO_3_, 7 MgCl_2_, 0.5 CaCl_2_, 2.5 KCl, 7 glucose, 3 pyruvic acid, and 1.3 ascorbic acid, bubbled with carbogen (O_2_ 95%, CO_2_ 5%). Coronal rat or mouse brain slices (300 µm) were prepared from the forebrain. Briefly, following transcardial perfusion and decapitation, the rat brain was exposed dorsally and was cut vertically between the cerebral cortex and cerebellum. The brain, now devoid of the cerebellum and brainstem, was quickly removed from the cranial cavity and immersed in ice-cold cutting solution for 1–2 min. Coronal slices containing the medial PFC including the rostral anterior cingulate cortex and prelimbic cortex (Bregma 2.28 ∼ 3.72 mm in rats, according to Paxinos and Watson 1998; and Bregma 1.10 ∼ 1.98 mm in mice, Paxinos and Franklin 2001) were obtained using a vibratome Leica VT1000S (Richmond Hill, Ontario) in the same solution. Brain slices were transferred to a standard extracellular solution (see below) to settle down at room temperature for at least 1 h before recording.

Brain slices were placed in a recording chamber mounted on the stage of an upright microscope Axioskop (Zeiss, Oberkochen, Germany) equipped with a 63X water immersion objective and differential contrast optics. A near-infrared charged-coupled device camera (Sony XC-75) was used to visualize the neurons. Brain slices were stabilized using a U-shaped stainless steel anchor with Lycra® threads at 1.5 mm spacing (Warner Instruments, Hamden, CT). Pyramidal neurons located in layer 2/3 or outer layer 5 with a typical pyramidal shape were chosen for electrophysiological recording. Slice perfusion was driven by gravity at a speed of 0.5–1 ml/min. The temperature of the perfusion solution was maintained at 32–33°C using a TC-324B temperature controller (Warner Instruments, Hamden, CT). Patch pipettes (5–7 MΩ) were pulled on a Brown Flaming puller (Model P-97, Sutter Instruments, Novato, CA) using borosilicate glass electrodes. Tight seals (∼5 GΩ) were obtained by applying constant negative pressure on the patch pipette. Electrical signals were amplified using an Axopatch 200B amplifier (Axon Instruments, Molecular Devices, Sunnyvale, CA), low-pass-filtered at 5 kHz, digitized at 10 kHz via a Digidata 1322A interface (Axon Instruments) and stored on a Pentium computer using the pClamp 9.2.1.8 software (Axon Instruments) for off-line analysis. In the present study, all cells were quiescent at rest and had a resting membrane potential ranging from −60 to −80 mV (median −73 mV). Cells with a resting membrane potential more positive than –55 mV were discarded. In current clamp recordings, the holding current was around 0 pA and slightly adjusted to obtain a membrane potential of –60 mV during drug testing. Series resistance (in most cases <20 MΩ) was not compensated. Input resistance was assessed by injecting negative current pulses (−60 to −150 pA, 500 ms) at −60 mV. After the whole cell patch was formed, a depolarizing current pulse (∼100–150 pA, duration 1 or 2 s) was applied before drug administration to induce repetitive spiking during the pulse (control) and after application of NE or carbachol (CCh) so that a long-lasting sustained repetitive spiking, which outlasts the stimulation pulse (persistent firing), was induced. This depolarization current pulse was sufficient to induce persistent firing following drug administration and was kept constant for every NE-responsive cell before and after drug testing. The induced persistent firing could last for a significant period of time (more than 10 min) after induction, but in almost all cases, it was terminated by injecting a hyperpolarizing current 2 or 3 minutes after induction. The firing frequency is defined as the average spiking frequency within 20 s after the depolarizing current pulse. The plateau potential refers to the average membrane potential during long lasting persistent firing or during short (<20 s) sustained firing (ADP with superimposed action potentials). The amplitude of plateau potentials reflects the difference between the mean membrane potential (mV) measured at baseline within one minute (before the pulse) and the mean membrane potential measured during the steady-state phase of persistent firing (excluding action potentials and afterhyperpolarizing potentials). In voltage clamp experiments for recording excitatory postsynaptic currents, the membrane potential was held at –70 mV (approximate reversal potential of inhibitory postsynaptic currents) and series resistance was compensated (>70%). To record the hyperpolarization-activated cation current (Ih), the membrane voltage was held at –50 mV and followed by the application of voltage steps (duration 2.5 s) ranging from –50 mV to –110 mV, with increments of –10 mV, to neurons.

### Optogenetics

Male tyrosine hydroxylase TH::IRES-Cre knock-in mice (EM:00254; B6.129X1-Thtm1(cre)Te/Kieg; European Mouse Mutant Archive) [30] were housed in a temperature- and humidity-controlled (40–60%) room under a 12 h light/dark cycle. Mice were given food and water ad libitum. Experimental protocols were approved by McGill animal care committee and meet the guidelines of the National Institutes of Health guide for the Care and Use of Laboratory Animals. Cre-inducible recombinant adeno-associated viruses (AAV) [31], [32], [33], [34] were used to genetically target channelrhodopsin-2 (ChR2) expression to noradrenergic neurons in the locus coeruleus as described previously [33]. Double-floxed reverse EF-1α::ChR2(H134R)-eYFP and EF-1α::eYFP cassettes were packaged in AAV vectors and serotyped with AAV5 coat proteins to produce high-titer virus preparations (2×10^12^ genome copies/ml; viral vector core facility at the University of North Carolina). Eight- to 10-week-old male TH::IRES-Cre mice were anesthetized using isoflurane. AAV viruses were delivered stereotactically (from bregma: anterior-posterior, –5.45 mm; lateral, 1.28 mm; and dorsal-ventral, 3.65 mm) as described previously [33]. One microliter of purified double-floxed AAV:ChR2-eYFP or control AAV:eYFP virus was injected bilaterally slightly laterally to the locus coeruleus area. The location of the viral injection as well as ChR2(H134R)-eYFP expression were verified by examining eYFP fluorescence in the locus coeruleus in brainstem slices. All mice were singly housed after surgery and recovered for at least 3 weeks before electrophysiological experiments. Activation of ChR2 channels was evoked by light pulses (10 ms pulse width) delivered at 3 Hz for 5–10 min with a TTL-driven 473 nm blue laser (Laserglow Technologies, Ontario, Canada). Tonic firing of 3 Hz in noradrenergic neurons of the locus coeruleus is correlated with active wakefulness in the rat and primate [35], [36]. It appears that high-frequency, non-physiological stimulation (>5 Hz) causes behavioral arrest and a decrease in cortical NE release as assessed by microdialysis, suggesting depletion of NE in noradrenergic terminals. Furthermore, 3 Hz of optical stimulation causes effective sleep-to-wake transitioning and an increase in total wakefulness in behavioral rats [33]. Therefore, we chose to deliver 3 Hz optical stimulation and simultaneously monitor excitatory postsynaptic events in acute brain slices [33], [36].

### Drugs and Solutions

All drugs were purchased from Sigma (Oakville, Ontario, Canada) except 6-methyl-2-(phenylethynyl)pyridine (MPEP), CGP 54626, 4-[1-Hydroxy-2-[(1-methylethyl) amino]ethyl]-1,2-benzenediol hydrochloride (isoproterenol) and ZD7288 which were purchased from Tocris (Tocris Bioscience, Ellisville, MO), propranolol from EMD biosciences and TTX from Alomone Labs (Jerusalem, Israel). Carbachol, clonidine, (S)-3,5-dihydroxyphenylglycine hydrate (DHPG), isoproterenol, norepinephrine, phenylephrine, prazosin, yohimbine, propranolol, TTX and ZD7288 were dissolved in water whereas MPEP and CGP 54626 were dissolved in dimethyl sulfoxide (DMSO). All drugs were freshly diluted from stock to the desired concentrations before the experiments; the final concentration of DMSO did not exceed 0.1%. In rats, the standard extracellular solution in current clamp experiments contained (in mM): 125 NaCl, 2.5 KCl, 1.6 CaCl_2_, 2 MgCl_2_, 25 NaHCO_3_, 1.25 NaH_2_PO_4_, 3 pyruvic acid, 1.3 ascorbic acid, 10 glucose, 2 kynurenic acid and 0.1 picrotoxin. Kynurenic acid and picrotoxin were used to block ionotropic synaptic transmission mediated by NMDA, non-NMDA (AMPA and kainate) and GABA_A_ receptors, respectively. pH was maintained at 7.4 by constant bubbling with carbogen (95% O_2_, 5% CO_2_). The intracellular solution in current clamp recordings contained (in mM): 120 Kgluconate, 20 KCl, 2 MgCl_2_,0.2 EGTA, 10 HEPES, 7 di-tris phosphocreatine, 4 Na_2_-ATP and 0.3 Tris-GTP. pH was adjusted to 7.3 with KOH. The extracellular solution used in voltage clamp recordings of spontaneous excitatory postsynaptic currents (sEPSCs) in rats was the same as in current clamp recordings except that kynurenic acid was omitted. BaCl_2_ (0.2 mM) was added to the extracellular solution in voltage clamp experiments to record the hyperpolarization-activated current (Ih). The intracellular solution used in voltage clamp recordings is the same as the one used in current clamp recordings. In optogenetic experiments in mice, the extracellular solution for both voltage and current clamp recording contained (in mM): 124 NaCl, 3 KCl, 1.6 CaCl_2_, 1.8 MgSO_4_, 26 NaHCO_3_, 1.25 NaH_2_PO_4_, 10 glucose, 0.1 picrotoxin pH 7.4. The intracellular solution used is the same as in rats.

### Data Analysis

Electrophysiological data were analyzed using Clampfit 9.2.1.8 (Axon Instruments) and Origin 6.0 (Microcal Software, North Hampton, MA). Values are expressed as means ± SEM. Synaptic event (sEPSCs) data collected in 120 s episodes from voltage clamp recordings before and after drug administration or light stimulation were analyzed using the Mini Analysis Program (Synaptosoft Inc NJ USA). The threshold for event detection was set at 10 pA. Cumulative probability curves were compared using the Kolmogorov-Smirnov two-sample test. Two-sample paired *t*-tests were used to compare mean values obtained before and after drug administration in the same neurons. Two-sample independent *t*-tests were used for comparison between 2 independent groups. One-way analysis of variance with Bonferroni correction was used for comparison of multiple independent groups. Differences were considered statistically significant when p<0.05.

## Results

### Norepinephrine Induces Persistent Firing in the PFC via α1 Adrenoceptors

**Figure 1:**
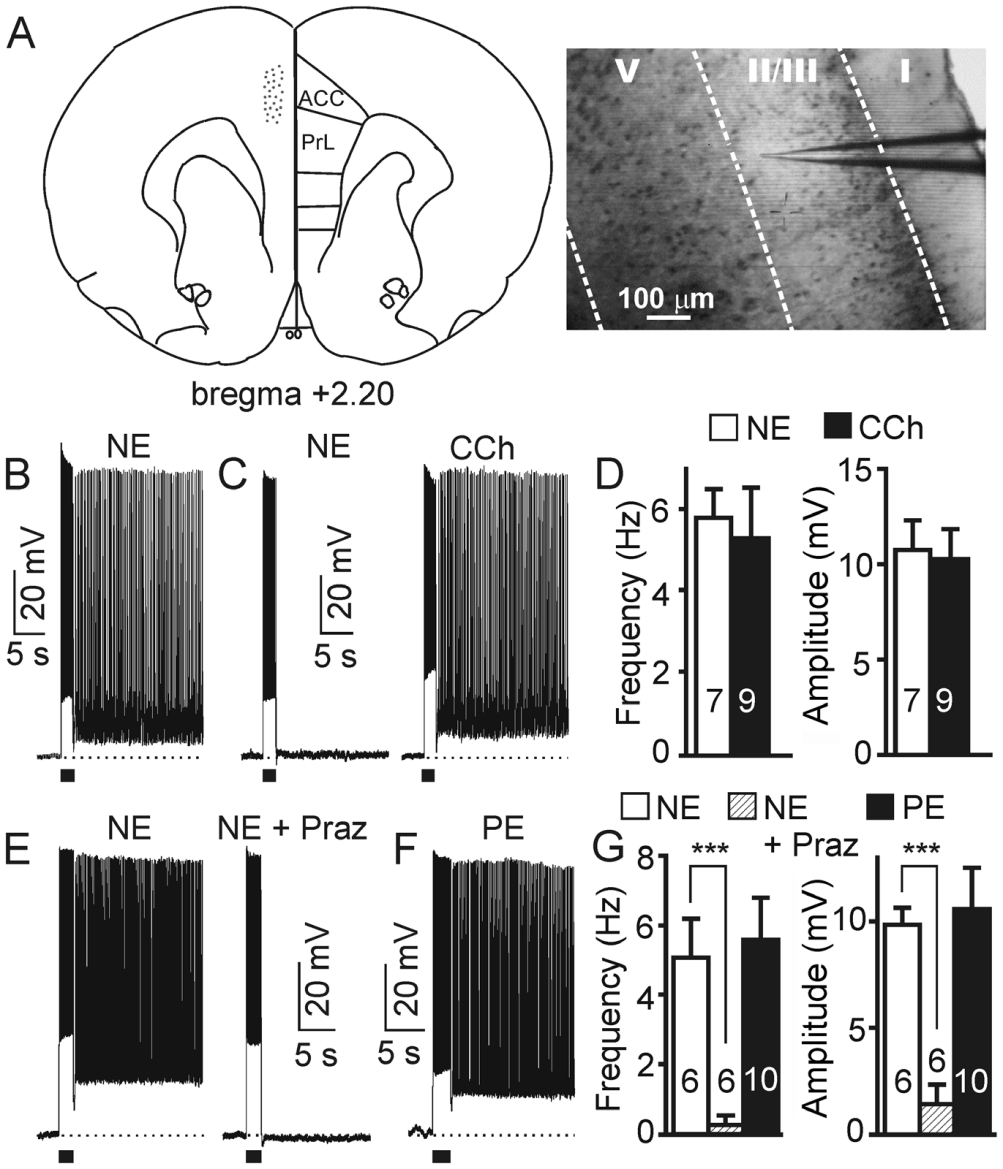
NE induces persistent firing in pyramidal neurons of prefrontal cortex via activation of α1 adrenoceptors. (A) Left: Schematic diagram of a coronal section of rat brain showing the recorded area (dots) in the prefrontal cortex including the rostral anterior cingulate cortex (ACC) and prelimbic cortex (PrL). Adapted from Paxinos and Watson (1998). Right: Typical video image of a brain slice showing the recording site (with a patch electrode) at layer 2/3 of the ACC subdivision of the prefrontal cortex. (B) Sample trace of NE (10 µM)-induced plateau potential and persistent firing following a depolarizing current pulse in a pyramidal neuron. (C) In another group of neurons, NE failed to induce neuronal responses (non-responsive cell, left panel). However, application of carbachol (CCh 10 µM) evoked persistent firing in the same neurons after NE washout (right panel). (D) Quantification of frequency and amplitude of plateau potential of the NE (10 µM, n = 7) and CCh (10 µM, n = 9) effects. (E) NE-evoked persistent firing (NE) is blocked by the selective α1 adrenoceptor antagonist prazosin (2 µM, NE+Praz, n = 6). (F) The selective α1 adrenoceptor agonist phenylephrine (PE, 10 µM, n = 10) also induced NE-like persistent firing in pyramidal neurons of the prefrontal cortex. (G) Quantification of frequency and amplitude of plateau potential of the prazosin and phenylephrine effects shown in E and F. Values are mean ± SEM. *** p<0.001. The dashed line in panel B, C, E and F represents the membrane potential at −60 mV. The short horizontal bar (2 s) underneath the recording trace represents the current pulse stimulation. The description on dashed line and short horizontal bar also applies to Figures 2, 3, 5, 6, 7 and 8.

Some neurotransmitters and neuromodulators have been shown to induce the generation of persistent firing in cortical pyramidal neurons via Gq-coupled metabotropic receptors (e.g., muscarinic M1, mGluR5) [14]–[19], [22], but the exact role of norepinephrine (NE) on the firing activity of the PFC is not clearly defined. We observed that application of NE (10 µM) induced long-lasting persistent neuronal firing following a depolarizing current pulse injection in ∼ 40% (57 out of 142) of the pyramidal neurons in superficial layers (layer 2/3 and outer layer 5) of the PFC (Figure 1A,B,D) in the presence of kynurenic acid and picrotoxin. We never observed persistent activity before application of NE (0 out of 142). Interestingly, application of the muscarinic receptor agonist carbachol (CCh) (10 µM) effectively induced persistent firing in all neurons tested including those that did not respond to NE (Figure 1C,D; n = 9), indicating that the cellular components required for persistent activity are present in these neurons non-responsive to NE. The NE response, characterized by a sustained plateau potential, does not desensitize within 1 hr of perfusion since persistent firing of approximately the same amplitude can be further induced multiple times after termination by hyperpolarization in the continuous presence of NE. Therefore, same cell controls were used in all experiments (before and after drug pairs) and, except when indicated otherwise, only neurons with induced long-lasting persistent firing were used for drug testing and analysis.

Application of the α1 adrenoceptor antagonist prazosin (2 µM) completely blocked the response evoked by NE (Figure 1E,G; n = 6). Furthermore, application of the selective α1 adrenoceptor agonist phenylephrine (PE, 10 µM) also induced long-lasting persistent firing in pyramidal neurons of the PFC (Figure 1F,G; n = 10, 35% cells tested), confirming the involvement of α1 adrenoceptors in the NE-evoked persistent response.

### α2 Adrenoceptor Activation Facilitates NE-induced Persistent Firing

**Figure 2:**
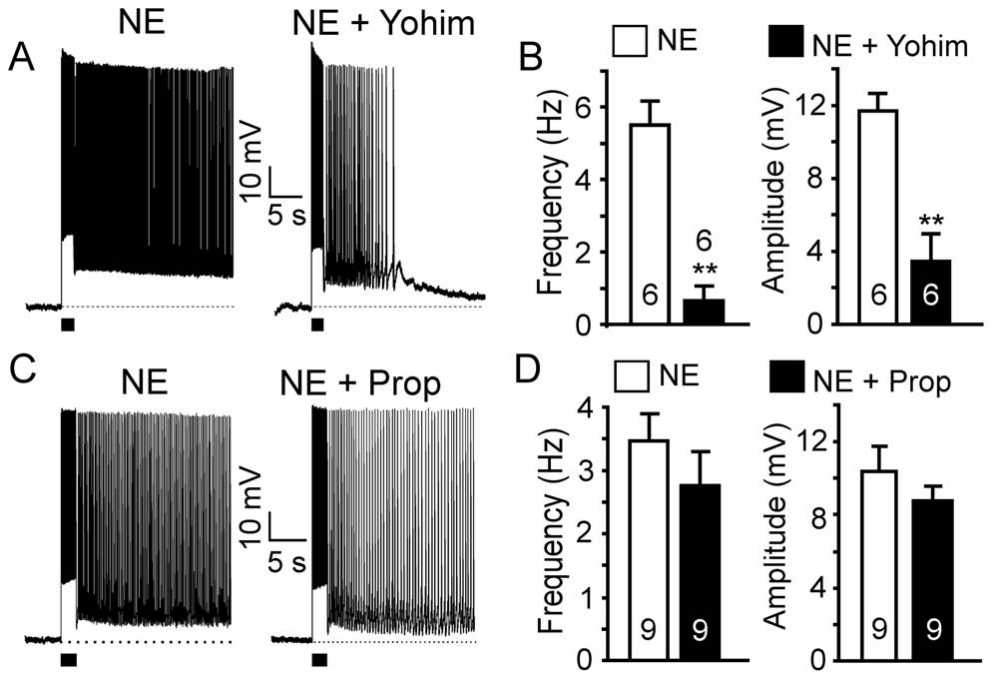
α2 adrenoceptors contribute to NE-induced persistent firing. (A) NE-induced persistent firing was partially suppressed by the selective α2 adrenoceptor antagonist yohimbine (Yohim, 10 µM; n = 6). (C) NE-induced persistent firing was not affected by the βadrenoceptor antagonist propranolol (Prop, 10 µM; n = 9). (B) and (D) Quantitative effects of yohimbine and propranolol on firing frequency and amplitude of plateau potentials. Values are mean ± SEM. ** p<0.01.

To test the contribution of α2 adrenoceptor activation in NE-evoked persistent firing, we applied the α2 antagonist yohimbine in the perfusion medium. Yohimbine (10 µM) partially inhibited persistent firing evoked by NE (10 µM) (Figure 2A). Both the firing frequency and the amplitude of the plateau potentials were significantly decreased by yohimbine (frequency from 5.5±0.7 Hz to 0.6±0.4 Hz, p<0.01, n = 6; amplitude from 11.7±0.9 mV to 3.5±1.5 mV, p<0.01, n = 6; Figure 2B). Interestingly, application of the α2 agonist clonidine (10 µM) did not induce persistent firing in pyramidal neurons (n = 10; not shown), suggesting that α2 adrenoceptor activation facilitates NE-induced persistent firing but does not initiate it.

Application of the pan-β adrenoceptor blocker propranolol (10 µM) did not have significant effects on the NE-induced persistent firing (frequency 3.5±0.4 Hz to 2.8±0.5 Hz, p>0.05, n = 9; amplitude from 14.0±1.4 mV to 8.8±0.8 mV, p>0.05, n = 9; Figure 2C,D) and the β adrenoceptor agonist isoproterenol (10 µM) did not induce persistent firing (n = 8, not shown). We conclude that, despite the fact that α2 adrenoceptors contribute to the modulation of persistent firing, neither α2 nor β adrenoceptors play an important role in the initiation of persistent neuronal firing in superficial pyramidal neurons of the PFC. Early reports suggested that β adrenoceptor activation has no significant effect on the working memory function of the PFC [37], [38]. More recent works described that activation of β1 adrenoceptors impairs working memory [39], whereas activation of β2 adrenoceptors has beneficial effects [40]. Because we did not observe any significant effects of pan-β adrenoceptor blockade on noradrenergic persistent firing, we did not investigate further the effects of different β adrenoceptor subtypes.

### α1 Adrenoceptor-Mediated Persistent Activity Depends on mGluR5 Activation

**Figure 3:**
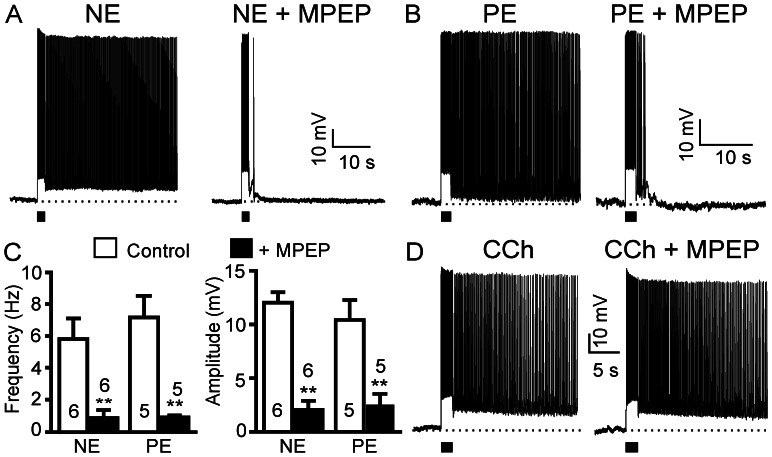
NE-evoked and α1 adrenoceptor-mediated persistent activity depends on mGluR5 activation. *(*A) and (B) NE- and the selective α1 adrenoceptor agonist phenylephrine (PE)-induced persistent firing were both blocked by the selective mGluR5 receptor antagonist MPEP (50 µM, n = 6 and n = 5, respectively). (C) Quantitative results of the frequency and the amplitude of plateau potentials from neurons exemplified in A and B. Values are mean ± SEM. ** p<0.01. (D) MPEP failed to block CCh-induced persistent firing (n = 4).

As a neuromodulator, NE could have either direct postsynaptic effects on pyramidal neurons by binding to postsynaptic adrenoceptors on the dendrites and/or soma of the cells or indirect effects through modulation of presynaptic neurotransmitter release. We have previously reported that glutamatergic persistent firing in the PFC is mediated by the metabotropic receptor mGluR5 [22], but not ionotropic glutamate receptors. Thus, we tested whether the NE effect was linked to mGluR-evoked responses. Indeed, application of the selective mGluR5 antagonist MPEP (50 µM) suppressed NE-induced persistent activity (Figure 3A). Both the firing frequency and the amplitude of plateau potentials were reduced to a minimum, strongly indicating an indirect effect of NE via release of glutamate and activation of metabotropic glutamate receptors (frequency from 5.8±1.3 Hz to 0.9±0.5 Hz, p<0.01, n = 6; amplitude from 12.0±0.9 mV to 1.9±0.8 mV, p<0.01, n = 6; Figure 3A,C). In agreement, the PE-induced and α1 adrenoceptor-mediated persistent firing was also blocked by MPEP in pyramidal neurons of the PFC (frequency from 7.2±0.4 Hz to 0.9±0.1 Hz, p<0.01, n = 5; amplitude from 10.5±1.8 mV to 2.5±1.0 mV, p<0.01, n = 5; Figure 3B,C). In contrast, within the same cells, the CCh-induced persistent firing was not affected by MPEP (n = 4; Figure 3D).

### Noradrenergic Persistent Response is Linked to Presynaptic Glutamate Release

**Figure 4:**
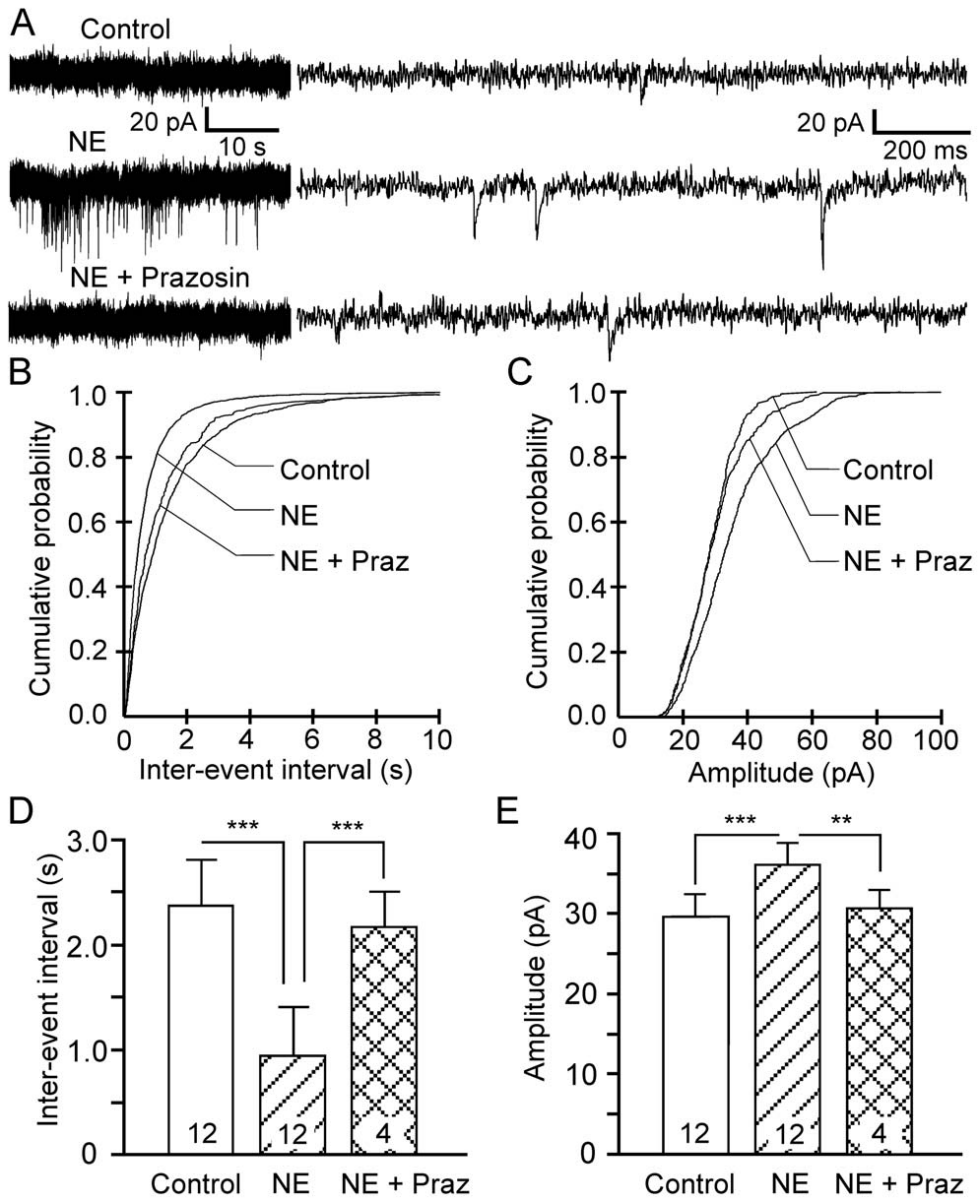
NE stimulates glutamate release in the prefrontal cortex. (A) Sample traces showing that NE (10–100 µM; n = 12) increases the frequency and amplitude of spontaneously occurring EPSCs (sEPSCs) in pyramidal neurons (Vh = −70 mV). Traces on the left and right are on different time scales. Prazosin (2 µM, n = 4) blocked this facilitatory effect. (B) and (C) Cumulative probability distribution curves for inter-event interval (reciprocal of frequency) and for amplitude of sEPSCs showing the NE-induced increase in frequency and amplitude of sEPSCs. Prazosin significantly suppressed the NE effect. (D) and (E) Quantitative effects of NE and NE+prazosin on inter-event intervals and amplitude of sEPSCs. Values are mean ± SEM. ** p<0.01; *** p<0.001.

To investigate a possible presynaptic site of action of NE on axons or axon terminals in α1 adrenoceptor-mediated persistent firing, we recorded glutamatergic excitatory synaptic events using voltage clamp in superficial pyramidal neurons of the PFC in the presence of the GABA blocker picrotoxin but with ionotropic glutamatergic transmission intact. Bath application of NE (10–100 µM) resulted in significant changes in the frequency and amplitude of the spontaneous excitatory postsynaptic currents (sEPSCs) (n = 12; shown at 10 min after NE, Figure 4A). NE shifted the cumulative probability distribution curve of inter-event intervals to the left while the curve of the amplitude was shifted to the right, indicative of increases in frequency and amplitude, respectively (Figure 4B,C). The average inter-event interval at 10 min was reduced from 2.4±0.4 s to 0.9±0.2 s (p<0.001, n = 12; Figure 4D). The average amplitude of the sEPSCs was increased from 30.6±2.9 pA to 35.9±2.9 pA (n = 12, p<0.001; Figure 4E), indicating an involvement of postsynaptic adrenoceptors. Addition of the ionotropic glutamate receptor antagonist kynurenic acid (1 mM) suppressed the NE effect (not shown, n = 4), indicating that it is mediated by glutamate release and the activation of ionotropic glutamate receptors. Furthermore, the NE effect was also suppressed by application of the α1 adrenoceptor antagonist prazosin (2 µM) (Figure 4A; n = 4). The inter-event interval and amplitude of sEPSCs were almost fully reversed to control levels after 10–15 min in the presence of prazosin (Figure 4B–E). NE might increase the frequency of sEPSCs by affecting the input resistance. We monitored the input resistance during the experiments and noticed that some neurons showed an increase in input resistance after NE application while others showed slight decreases or no change. We nonetheless observed a consistent increase in the measured frequency of sEPSCs. Thus, to confirm a presynaptic effect of NE, we averaged the EPSCs with large amplitudes (∼ 50–100 pA), which are supposed to be generated near the soma, and the EPSCs with smaller amplitudes (20–25 pA), which are supposed to come from remote dendritic locations, from 3 neurons that displayed no significant changes in input resistance. The falling phases of the EPSCs were fitted with a single exponential function. We observed no significant difference in the time to decay (peak to end) between the large and smaller average EPSCs in all three neurons (p = 0.0887, p = 0.2688, and p = 0.5928, respectively) indicating that, in these neurons, input resistance is not the reason why smaller EPSCs are detected. Thus, the detection of more sEPSCs is due to an increase in the frequency of sEPSCs. To further confirm a presynaptic effect of NE and to check for a possible contribution of postsynaptic α1 receptor on persistent firing, we recorded miniature EPSCs (mEPSCs) with TTX (1 µM) in the perfusion solution. We found that the α1 adrenoceptor agonist phenylephrine (10 µM) increased the frequency of mEPSCs consistently in 5 cells (control: 1.65±0.4 Hz, phenylephrine: 2.12±0.4 Hz, p = 0.0166, n = 5, data not shown). There was a positive trend but no significant effect of phenylephrine on the amplitude of mEPSCs (control: 13.61±0.9 pA, phenylephrine: 15.89±2.4 pA, p = 0.2240, n = 5, data not shown). Interestingly we did not observe any significant changes in both frequency and amplitude of mEPSCs in 2 cells after phenylephrine application, confirming the existence of a population of pyramidal neurons non-responsive to NE or phenylephrine. Therefore, our findings support a TTX-insensitive presynaptic site of action of NE through α1 adrenoceptors in a subpopulation of superficial pyramidal neurons of the PFC.

**Figure 5:**
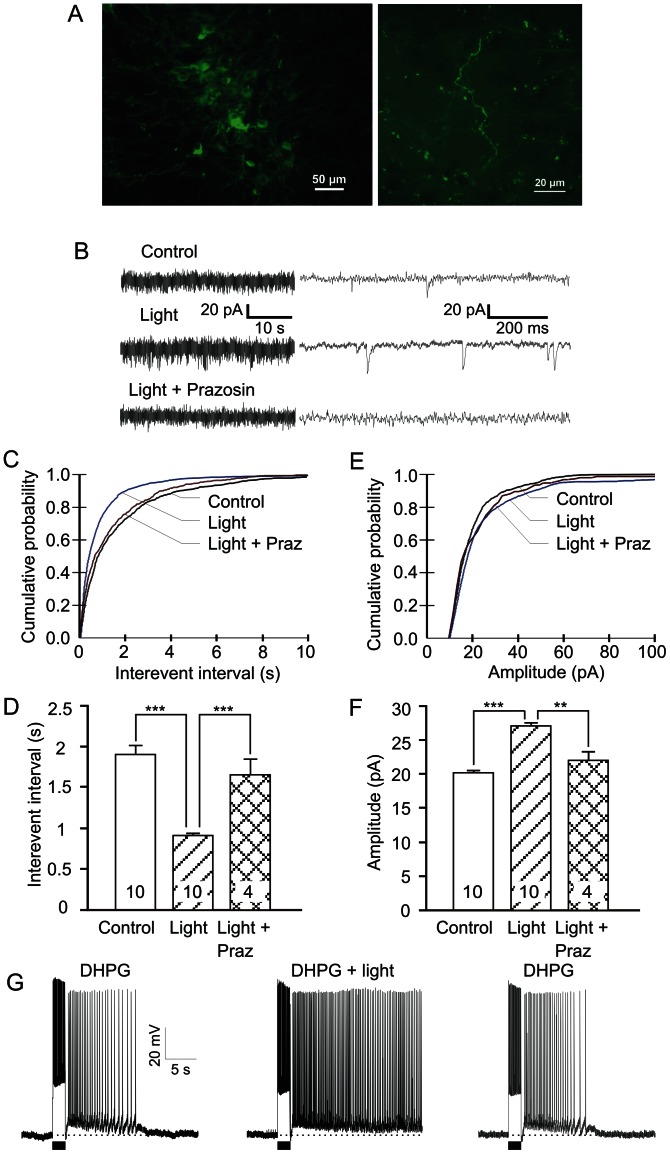
Optogenetically-evoked NE release enhances persistent firing. (A) Micrographs of AAV-infected and fluorescent eYFP-positive, ChR2-expressing noradrenergic neurons in the locus coeruleus (left) and their axonal fibers with varicosities in the medial prefrontal cortex of TH::Cre transgenic mice (right). (B) Representative traces showing that blue light (473 nm, 3 Hz, 10 ms, >10 mW/mm^2^, n = 10) increased both the frequency and amplitude of sEPSCs in pyramidal neurons (Vh = −70 mV). Traces on the left and right are on different time scales. Prazosin (2∼10 µM, n = 4) blocked this excitatory effect. (C) and (E*)* Cumulative probability distribution curves for inter-event interval and amplitude of sEPSCs showing the increase in the frequency and amplitude of sEPSCs. Prazosin significantly suppressed the ChR2-induced effects. (D) and (F), Quantitative effects of blue light and the antagonistic effect of prazosin on inter-event intervals and amplitude of the sEPSCs. Values are mean ± SEM. ** p<0.01; *** p<0.001. (G) Enhancement of DHPG-evoked response by optically-induced NE release and recovery.

To verify if activation of noradrenergic fibers originating from the locus coeruleus could induce similar effects as exogenous NE application in brain slices, we used an optogenetic approach. We expressed ChR2-eYFP in noradrenergic neurons and their fibers that project to the PFC by transducing the locus coeruleus of (TH)::IRES-Cre knock-in mice with Cre-inducible adeno-associated virus as previously shown [33]. Three weeks post-injection, a strong eYFP fluorescence was observed in the cell bodies of the locus coeruleus neurons in brainstem slices (Figure 5A). A high density of fluorescent axonal fibers and varicosities was also clearly visible in prefrontal brain slices, indicating the expression of ChR2 (Figure 5A). Activation of the ChR2-expressing noradrenergic fibers in acute brain slices of the PFC upon 3 Hz optical stimulation (see “Materials and Methods”) significantly enhanced excitatory postsynaptic activity in superficial pyramidal neurons in the absence of kynurenic acid in the perfusate (Figure 5B; n = 10). The cumulative probability distribution curve of inter-event intervals at 10 min was shifted to the left upon optical stimulation with a mean inter-event interval reduced from 1.9±0.1 s to 0.9±0.1 s (p<0.001, n = 10, Figure 5C, 5E). This increase in frequency of sEPSCs was suppressed by the selective α1 antagonist prazosin (2∼10 µM, Figure 5B–D; n = 4). The amplitude of sEPSCs was also affected by optical stimulation as shown by the right-shifted cumulative probability curve and the increase in the mean amplitude of sEPSCs from 20.2±0.4 pA to 27.0±0.5 pA (p<0.001, n = 10, Figure 5B,E,F). This increase in sEPSC amplitude was significantly suppressed by prazosin (2∼10 µM, n = 4, Figure 5B,E,F), confirming a postsynaptic role for α1 adrenoceptors. Therefore, release of endogenous NE evoked by optogenetic stimulation of prefrontal noradrenergic fibers could effectively increase the frequency of glutamate release via the activation of α1 adrenoceptors. However, no significant changes in postsynaptic activities were observed in eYFP control mice (n = 2) or eYFP-ChR2 mice without blue light stimulation (n = 5, not shown). To further test if release of endogenous NE can contribute to persistent activity, we recorded the activity of pyramidal neurons in current clamp under optical stimulation. We observed typical afterdepolarizations 5 min after optical stimulation (n = 5, not shown). When the pyramidal neurons were primed with threshold concentration (2 µM) of the group I metabotropic receptor agonist DHPG, optically-evoked NE release triggered long-lasting persistent firing (n = 3, Figure 5G).

### NE-evoked Persistent Firing is Modulated by α2 Adrenoceptor-linked HCN Channels

Another question we pondered was why NE failed to evoke persistent firing in a population of pyramidal neurons in superficial layers of the PFC. It has been reported that presynaptic GABA_A_ receptors mediate presynaptic inhibition of glutamate release from primary muscle afferents in the spinal cord, but have a facilitatory effect on neurotransmitter release in the brainstem, cerebellum and hippocampus [41], [42], [43]. It is possible that presynaptic GABA_A_ modulation of glutamate release affects persistent firing. Therefore we performed further experiments to test if NE-induced persistent firing is sensitive to blockade of fast GABAergic transmission. We found that NE induces persistent firing in pyramidal neurons in the absence of picrotoxin (n = 3, data not shown), as observed in the presence of picrotoxin, suggesting that GABA_A_ modulation of glutamate release does not play a major role. The failure of NE to induce persistent firing in a population of cells could also result from tonic GABAergic inhibition mediated by GABA_B_ receptors. To test this possibility, we added the selective GABA_B_ receptor antagonist CGP 54626 in the perfusate. Application of CGP 54626 (10 µM) together with NE did not unmask any excitatory effect in the NE non-responsive cells (n = 4; not shown), excluding a major role for metabotropic GABAergic modulation in the expression of NE-evoked persistent responses in pyramidal neurons.

**Figure 6:**
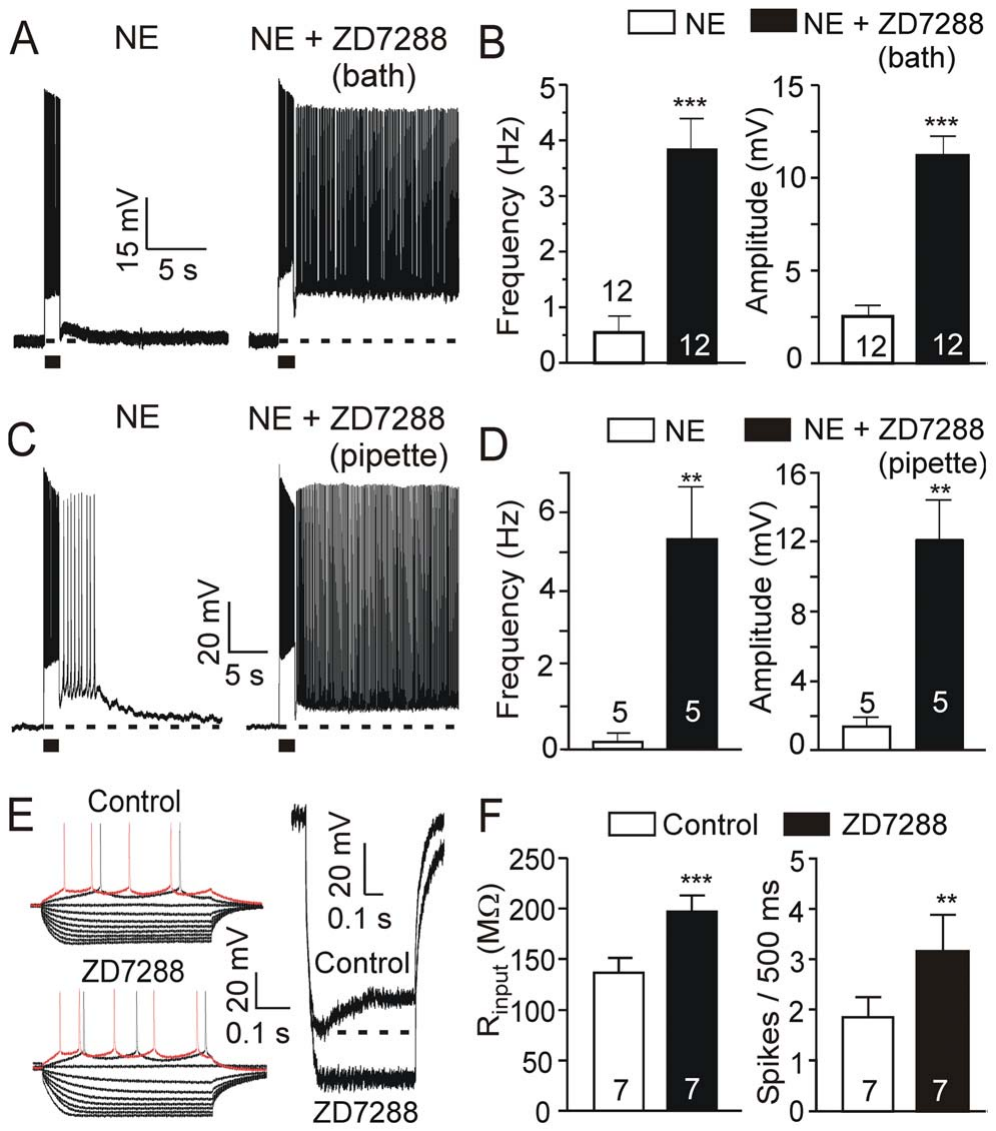
Noradrenergic responses are modulated by postsynaptic HCN channel activity. (A) A typical trace from a pyramidal neuron showing minimal response to bath application of NE (10 µM) (left panel). Co-application of NE and the selective HCN channel blocker ZD7288 (30 µM) in the bath solution in the same neuron significantly enhanced the NE-induced response (n = 12, right panel). (B) Quantitative effects of bath ZD7288 on firing frequency and amplitude of plateau potentials of NE-evoked response. (C) A threshold dose of NE (2.5 µM) induced afterdepolarization and short sustained firing (Left, without ZD7288, n = 5). In another group of neurons with ZD7288 in the recording pipette, NE induced strong persistent firing (Right, ZD7288 in pipette, n = 5). (D) Quantitative effects of intracellular ZD7288 on the firing frequency and amplitude of plateau potentials. (E) Sample traces of current step-evoked membrane potential responses before (control) and after ZD7288 application showing an increase in voltage responses (response to 40 pA current step shown in red) (left panel, n = 7). Current step stimulation from -140 pA to a maximum of 40 pA with 20 pA increments. Right: Membrane potential response to a -140 pA current step in control and in the presence of ZD7288. Note that the depolarizing membrane potential sag, a characteristic feature of HCN channel activation, was completely suppressed by ZD7288. (F) Input resistance (calculated at −200 pA) and number of spikes during the 40 pA current pulse (500 ms) before and after ZD7288 administration (n = 7). Values are mean ± SEM. ** p<0.01; *** p<0.001.

Previous reports have demonstrated that HCN channels are expressed on dendritic spines of pyramidal neurons in the PFC and that blockade of HCN channels via α2 adrenoceptor activation enhances recurrent network interactions in ferret PFC slices and increases delay-related firing for preferred directions in behavioral monkeys [44]. To test if the intrinsic persistent firing of rat medial PFC pyramidal neurons is modulated by HCN channel activity, we examined the effect of the selective HCN channel blocker ZD7288. Bath application of ZD7288 (30 µM) alone did not induce persistent firing (n = 10, not shown). However, treatment with ZD7288 enhanced the persistent response of a population of pyramidal cells to NE (10 µM): both the average firing frequency and the amplitude of plateau potentials of NE-evoked responses were significantly increased (frequency from 0.6±0.3 Hz to 3.8±0.6 Hz, p<0.001, n = 12; amplitude from 2.5±0.6 mV to 11.2±1.1 mV, p<0.001, n = 12, Figure 6A,B). Interestingly, besides the enhancement of persistent firing by ZD7288 in 6 of the 12 cells tested, the other 6 non-responsive cells showed strong persistent firing in response to NE following addition of ZD7288 (Figure 6A). The fact that blockade of HCN channels converted non-responsive neurons into neurons with persistent firing indicates that differential adrenoceptor expression and/or distinct intrinsic neuronal properties (e.g., HCN expression levels) likely account for the different responses to NE [45].

Recently, Huang and coll. [46] have shown that, with ZD7288 applied intracellularly through the patch pipette, bath application of ZD7288 increases the frequency, but not the amplitude, of mEPSCs compared to control (ZD7288 in pipette only). This indicates that presynaptic HCN channels regulate glutamate release and that the diffusion of ZD7288 from postsynaptic to presynaptic sites of action is limited within the recording time period (15–20 min). Therefore, we added the HCN channel blocker ZD7288 (30 µM) in the recording pipette. Intracellularly applied ZD7288 enhanced neuronal responses to a threshold dose of NE (2.5 µM, n = 5, compared to control, a separate group of neurons, n = 5; Figure 6C,D) to a similar extent as bath applied ZD7288 (Figure 6A,B), indicating that the NE-evoked persistent firing is mainly mediated by blockade of postsynaptic HCN channels. Furthermore, we applied hyperpolarizing and depolarizing current pulses to superficial pyramidal neurons through the recording electrode and measured membrane input resistance as well as neuronal excitability (Figure 6E,F). Application of ZD7288 (30 µM) completely eliminated the current pulse-evoked depolarizing membrane potential sag typically caused by HCN channel-mediated inward Ih currents (n = 7, Figure 6E right panel). Moreover, ZD7288 increased the membrane input resistance (from 136.7±15.2 MΩ to 197.7±16.5 MΩ, p<0.001, n = 7, Figure 6E and Figure 6F left panel), as well as the cell responsiveness (number of action potentials) to depolarizing current pulses (from 1.8±0.4 spikes/500 ms to 3.2±0.7 spikes/500 ms, p<0.01, n = 7, Figure 6E left panel, 6F right panel), indicating that blockade of postsynaptic HCN channels mediates the enhancement of cell excitability and NE-evoked persistent firing.

**Figure 7:**
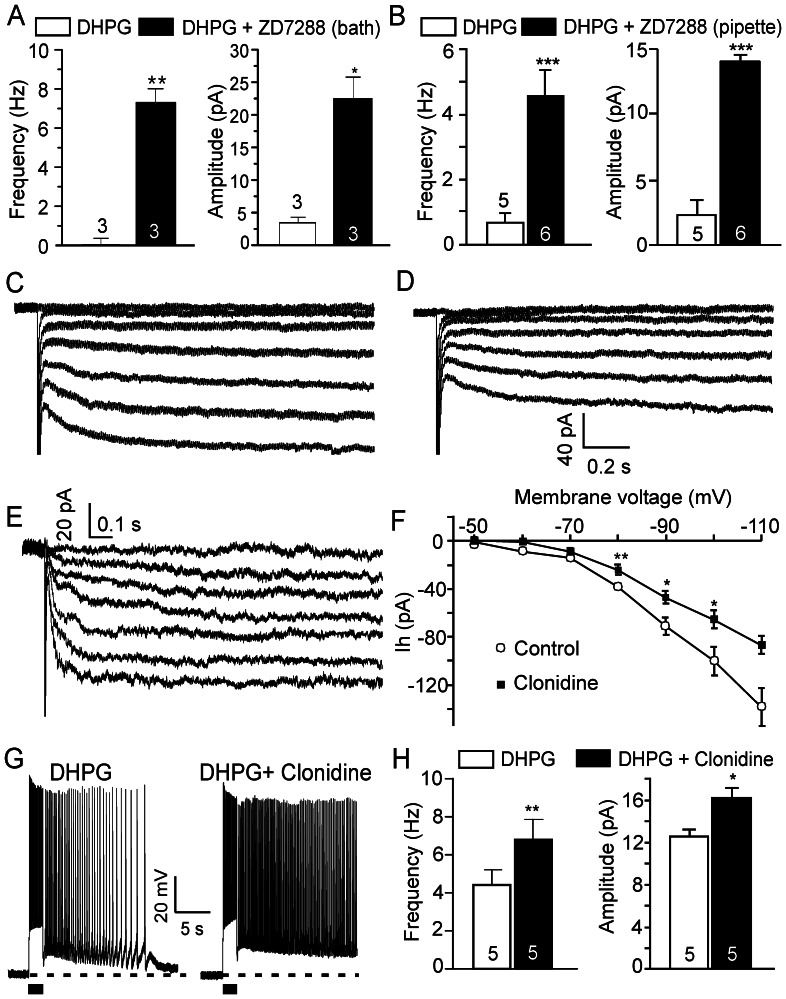
Glutamatergic persistent firing is enhanced by α2 adrenoceptor-mediated HCN channel inhibition. (A) and (B) Quantitative effects of bath and in-pipette ZD7288 (30 µM) on responses of PFC pyramidal neurons to a threshold dose of DHPG (2 µM). (C), (D) and (E) Sample traces showing clonidine-sensitive Ih currents (E) at different membrane voltages obtained by subtracting current responses to voltage steps after bath application of clonidine (10 µM) (D) from pre-drug control (C). Hyperpolarization-evoked Ih currents were inhibited by clonidine. Ih current responses were evoked by hyperpolarization voltage steps (duration 2.5 s) from −50 mV to –110 mV in 10 mV increments. (F) Steady state I-V relationship of the Ih current in the absence (Control) and presence of clonidine. Instantaneous currents were subtracted. Ih was inhibited by clonidine (10–20 µM) at different membrane voltages. (G) Representative traces showing that the response of prefrontal pyramidal neurons to threshold doses of DHPG (2 µM, left panels) are enhanced by co-application of the α2 adrenoceptor agonist clonidine (10 µM, n = 5, right panel). (H) Quantitative effects of clonidine on DHPG-induced firing frequency and amplitude of plateau potentials. Values in A, B, F and H are mean ± SEM. * p<0.05; ** p<0.01; *** p<0.001.

It has been reported that activation of α2 adrenoceptors inhibits HCN channels [47], [48]. As shown above, neither the α2 adrenoceptor agonist clonidine nor the selective HCN channel blocker ZD7288 induces persistent firing in pyramidal neurons, indicating that inhibition of HCN channels alone by NE (via α2 adrenoceptors) is not sufficient; it requires other components (e.g., glutamate released via α1 adrenoceptors) to induce persistent firing. To test the modulatory role of HCN channels on metabotropic persistent firing, we used the group I mGluR agonist DHPG to directly mimic α1 adrenoceptor-mediated release of glutamate. A threshold dose of DHPG (2 µM) induced slight responses in prefrontal pyramidal neurons in the absence of ZD7288; however, when we applied ZD7288 in the bath (Figure 7A) or in the pipette (Figure 7B, in a separate group of neurons from control), DHPG (2 µM) induced strong persistent firing (frequency from 0.1±0.3 Hz to 7.3±0.7 Hz, p<0.01, amplitude from 3.5±0.9 mV to 22.5±3.5 mV, p<0.05, n = 3 in bath; and frequency from 0.6±0.3 Hz to 4.6±0.7 Hz, p<0.0005, amplitude from 2.2±0.7 mV to 14.1±0.6 mV, p<0.0001, n = 5 in pipette, respectively), indicating that blockade of postsynaptic HCN channels indeed enhances glutamatergic persistent firing in the pyramidal neurons of the PFC.

The involvement of α2 adrenoceptor activation and HCN channel blockade in the enhancement of cell excitability and NE-induced persistent firing was further confirmed when we tested the effect of the α2 adrenoceptor agonist clonidine on HCN channel activity. We found that clonidine (10∼20 µM) suppressed hyperpolarization-evoked inward currents (Figure 7C–F, n = 7), indicating that the activation of α2 adrenoceptors inhibits Ih. Next, we used DHPG to mimic the α1 adrenoceptor-mediated release of glutamate and tested the effect of α2 adrenoceptor activation on metabotropic persistent firing. We found that the afterdepolarization or short persistent firing evoked by a threshold concentration of DHPG (2 µM) was converted by clonidine (10 µM) to bona fide long-lasting persistent firing (n = 5, frequency from 4.4±0.8 Hz to 6.8±1.1 Hz, p<0.01; amplitude from 12.6±0.7 mV to 16.2±0.9 mV, p<0.05; Figure 7G,H), indicating that both α1 and α2 adrenoceptors are required to synergistically mediate a strong noradrenergic persistent activity.

### NE Enhances Cholinergic Persistent Firing via α2 Adrenoceptors and HCN Channels

**Figure 8:**
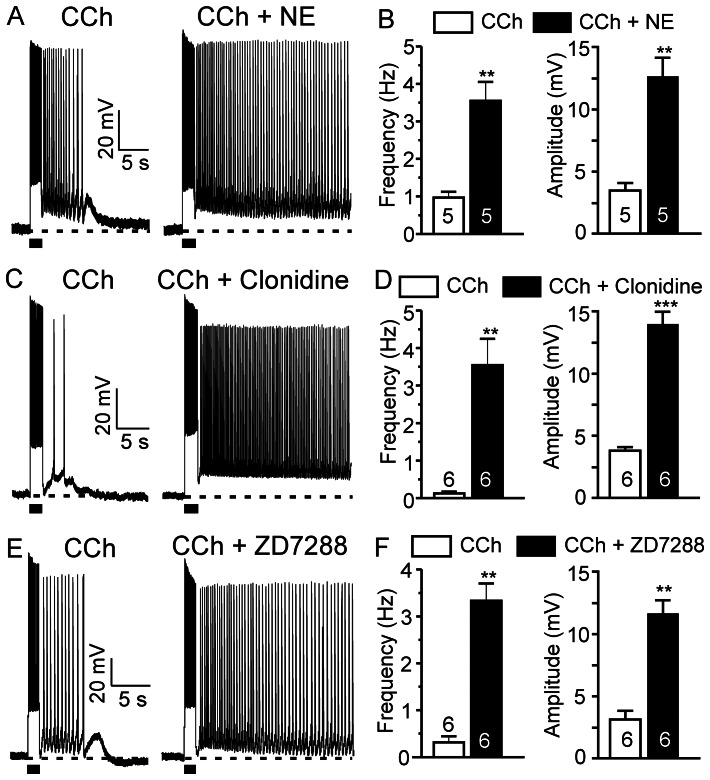
NE enhances cholinergic persistent neuronal firing via α2 adrenoceptor and HCN channel modulation. (A), (C) and (E) Threshold doses of CCh (0.5–2.5 µM) induce afterdepolarization potentials or short sustained firing (left panel). NE, clonidine and ZD7288 significantly enhanced the muscarinic response in pyramidal neurons of the prefrontal cortex (right panel). (B), (D) and (F) Quantitative effects of NE (n = 5), clonidine (n = 6) or ZD7288 (n = 6) on CCh-induced firing frequency and amplitude of plateau potentials. Values are mean ± SEM. ** p<0.01. *** p<0.001.

Activation of muscarinic cholinergic receptors induces persistent firing in pyramidal neurons of the PFC [22]. Furthermore, this report demonstrated that both the muscarinic cholinergic and the mGluR5 receptors converge on the same downstream intracellular signaling pathway that drives persistent firing [22]. Knowing that presynaptic α1 adrenoceptor-mediated glutamate release leads to mGluR5 responses that are additive to muscarinic responses, we wanted to assess the contribution of α2 adrenoceptor-linked HCN channels in the noradrenergic modulation of cholinergic persistent firing. To this end, we first studied the response of pyramidal neurons to co-application of CCh and NE. We found that a low concentration of NE (2.5 µM) significantly enhanced the excitatory responses of pyramidal neurons to threshold doses of CCh (0.5–2.5 µM) (n = 5, frequency from 0.9±0.2 Hz to 3.5±0.5 Hz, p<0.01; amplitude from 3.5±0.6 mV to 12.6±1.5 mV, p<0.01; Figure 8A,B).

To further pinpoint the mechanism of the noradrenergic modulation of cholinergic persistent firing, we tested the effects of α2 adrenoceptor activation or blockade of HCN channels on muscarinic responses. Threshold doses of CCh alone only induced short afterdepolarizations or short low frequency sustained neuronal firing in response to depolarizing current pulse injections. However, in the presence of clonidine (10 µM) or ZD7288 (30 µM), threshold doses of CCh evoked strong long-lasting persistent firing (Figure 8C–F). The average firing frequency and amplitude of plateau potentials were both dramatically increased in both conditions (clonidine: n = 6, frequency from 0.2±0.1 Hz to 3.6±0.7 Hz, p<0.01; amplitude from 3.8±0.3 mV to 13.9±1.0 mV, p<0.001. ZD7288: n = 6, frequency from 0.3±0.1 Hz to 3.3±0.4 Hz, p<0.01; amplitude from 3.2±0.7 mV to 11.6±1.1 mV, p<0.01) (Figure 8D and F, respectively). We conclude that the facilitatory effects of NE on the muscarinic persistent response can be mediated by the activation of α2 adrenoceptors and subsequent inhibition of HCN channels in pyramidal neurons of the PFC.

## Discussion

### NE-evoked Persistent Firing Depends on α1 Adrenoceptors

Immunohistochemical studies have documented dense noradrenergic fibers in the superficial layers of the neocortex [49], [50]. Autoradiographic ligand binding studies demonstrated that α1 adrenoceptor binding sites are abundant in superficial layers of the neocortex [51], [52]. Recent studies combining immunocytochemistry and electron microscopy have shown the expression of α1 adrenoceptors in axons or presynaptic terminals of excitatory neurons in the visual cortex [53], striatum and midbrain [54]. In the present study, we report that the NE-evoked persistent responses are completely blocked by the α1 antagonist prazosin while the selective α1 agonist phenylephrine induces persistent firing. Furthermore, the excitatory postsynaptic activity evoked by bath or endogenous NE was nearly completely abolished by prazosin, indicating the involvement of α1 adrenoceptors. In situ hybridization histochemistry has revealed significant α1 mRNA signals in thalamic nuclei and layers 2–5 neurons of the cerebral cortex [55], [56]. These α1 adrenoceptor-expressing neurons in the thalamus and the cerebral cortex projecting to layer 2/3 of the PFC are the probable sites of action of NE.

### Noradrenergic Persistent Firing Requires mGluR5 Activation

We provide several pieces of evidence to support the notion that the NE effect is indirectly mediated by presynaptic glutamate release: first, both NE- and the selective α1 agonist phenylephrine-evoked persistent firing were blocked by the mGluR5 blocker MPEP; second, both bath-applied NE and endogenous NE from the locus coeruleus increased the frequency of the sEPSCs and third, bath-applied phenylephrine increases the frequency of mEPSCs (in the presence of TTX). The fact that persistent firing induced by a high concentration of NE (100 µM, n = 3) is still blocked by the mGluR5 blocker MPEP indicates that mGluR activation is necessary for NE-induced persistent firing. Our results are in agreement with previous reports showing that NE can increase the frequency of EPSCs through α1 adrenoceptor activation in the hypothalamus [57], [58] and layer 5 of the PFC [59]. The prazosin-sensitive increase in the amplitude of sEPSCs induced by both bath NE and endogenous NE release indicates the expression of functional postsynaptic α1 adrenoceptors. This suggests that the persistent response to NE could involve additive postsynaptic effects of α1 adrenoceptor and mGluR5 activation. This increase in amplitude of sEPSCs may also be the result of highly synchronized multivesicular glutamate release. Such synchronized multivesicular release has also been observed with other neuromodulators in various brain areas [60], [61]. The NE-induced glutamate release (recorded as kynurenic acid-sensitive EPSCs) could originate from synaptic terminals of adjacent neurons and/or from axon terminals of remote neurons making synaptic contacts with adjacent neurons.

Activation of Group I mGluRs induces afterdepolarizations and persistent firing in the PFC [19], [22]. Interestingly, in the present study, the endogenous NE released by activation of light-gated ChR2 expressed in fibers from the locus coeruleus induced typical afterdepolarizations and persistent firing in the presence of a threshold amount of the group I mGluR agonist DHPG, indicating that endogenous release of NE can effectively modulate mGluR5 signalling via an enhancement of glutamate release. It is conceivable that in awake conditions, moderate NE levels induce persistent firing in the PFC through presynaptic release of glutamate, thereby promoting working memory performances. Accordingly, it has been shown that the decrease in group I mGluR function in the PFC causes impaired working memory performance [62], [63]. Furthermore, blockade of mGluR5 by systemic MPEP impaired working memory in the rat [64], [65]. Therefore, normal working memory requires the coordinated release of both NE and glutamate as well as the intact functional mGluRs in the PFC [66].

### HCN Channels Modulate Noradrenergic and Cholinergic Persistent Firing

It has been reported that activation of α2 adrenoceptors inhibits HCN channels and enhances delay-related prefrontal firing and working memory performance [44], [48], [67]. In agreement, we observed at the cellular level that NE-evoked responses are enhanced by blockade of postsynaptic HCN channels. The blockade of postsynaptic HCN channels increases input resistance of superficial pyramidal neurons in the PFC and enhances NE-evoked neuronal activity. Furthermore, we observed that α2 adrenoceptor activation modulates HCN channel-mediated Ih current and the mGluR5-mediated afterdepolarization is enhanced by simultaneous blockade of HCN channels and co-activation of α2 adrenoceptors. Therefore, the NE-mediated persistent firing in the PFC requires both α1 and α2 adrenoceptor activation. NE not only initiates persistent firing via α1 adrenoceptors and presynaptic glutamate release but also enhances it via α2 adrenoceptors, HCN channel modulation and increased postsynaptic excitability.

Activation of postsynaptic muscarinic receptors also induces persistent firing in pyramidal neurons of the PFC and other cortical areas [20], [22], [24], [68]. Activation of muscarinic receptors enhances synaptic summation in layer 5 pyramidal neurons of the PFC via blockade of several K^+^ conductances [69], [70]. Dendritic excitability of prefrontal pyramidal neurons has been shown to depend on the interaction between HCN, Kir2 and K_leak_ channels [69]. Our results indicate that both activation of α2 adrenoceptors and modulation of HCN channels can enhance CCh-evoked muscarinic responses suggesting that HCN channels are involved in the adrenergic modulation of cholinergic persistent firing. This HCN channel-dependent mechanism reveals a novel mode of crosstalk between cholinergic and adrenergic inputs in the PFC.

### Role of α1 Adrenoceptors in Working Memory

In the present study, we observed an indirect excitatory effect of NE on pyramidal neurons in the PFC through presynaptic α1 adrenoceptor-mediated glutamate release and postsynaptic mGluR5 activation. It has been proposed that high levels of NE inhibits prefrontal neuronal discharges and impairs working memory performance via postsynaptic α1 adrenoceptor-mediated PKC activation [29]. It is important to note that the α1 adrenoceptor-mediated excitatory effect in our study is mainly of presynaptic origin, whereas the inhibitory effect of α1 adrenoceptors in the above-mentioned work has been shown to be postsynaptic. There is increasing evidence for direct postsynaptic excitatory effects of several Gq-coupled metabotropic receptors (e.g., muscarinic, mGluR5, orexin receptor 1, 5-HT2A) on pyramidal neuron firing in various cortical regions [20], [22], [71], [72]. The Gq-coupled and PLC-linked persistent firing has been shown to require a non-selective cation current mediated by TRPC4 and/or TRPC5-containing channels [24]. Why postsynaptic Gq-coupled α1 adrenoceptors are not able to trigger plateau potentials and persistent firing will remain to be determined. Their surface density, subcellular localization or coupling to intracellular effectors could be critical parameters for their contribution to persistent responses. Why the activation of metabotropic receptors and associated Gq-PLC signaling can lead to an enhancement of neuronal firing whereas the activation of downstream PKC causes inhibition [29] is also unknown. It is possible that a strong and sustained activation of PKC desensitizes α1 adrenoceptors [73] or suppresses the mechanism required for the induction of persistent firing. Indeed, it has been reported that TRPC4/5 channels mediating metabotropic persistent firing are desensitized by diacylglycerol-induced activation of PKC [74], [75]. Following co-application of the muscarinic agonist CCh with 1-oleoyl-2-acetyl-sn-glycerol, an analogue of the PKC activator diacylglycerol, pyramidal cells gradually displayed an inhibition of the CCh-evoked persistent firing in acute entorhinal brain slices (unpublished data), indicating an inhibitory effect of PKC activation through TRPC4/5 channel desensitization. Therefore, in certain conditions, a direct inhibitory effect of postsynaptic α1 adrenoceptor activation could be caused by PKC-mediated desensitization of adrenoceptors and/or TRPC channels.

At the behavioural level, activation of α1 adrenoceptors is associated with attentional set shifting and cognitive flexibility in rats [76], [77], as well as with perseverative patterns of responses in stressed rats performing working memory tasks [28], [78]. There is a large increase in monoamine [79], [80] and glutamate [81] release in the PFC during stress. Moreover, overstimulation of α1 adrenoceptors was observed in patients with hypermnesia and anxiety-related syndromes such as PTSD [82]–[84]. Blockade of α1 adrenoceptors with prazosin has been used as a therapeutic strategy to reduce the reoccurrence of traumatic nightmares in patients with PTSD and to decrease relapse in patients with alcohol dependence [85]–[87]. Therefore, besides being involved in normal cognition, α1 adrenoceptor-mediated persistent firing is also likely associated with overexcitability and inappropriate activity in stress-induced pathological conditions, underlying the therapeutic effects of prazosin in the PFC.

**Figure 9:**
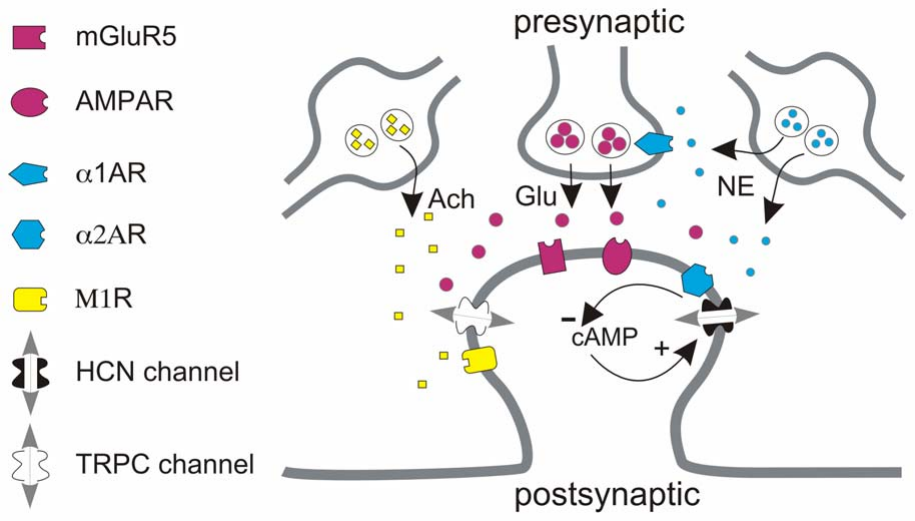
Molecular components involved in NE-induced persistent firing in superficial pyramidal neurons of prefrontal cortex. NE released from noradrenergic varicosities in the prefrontal cortex binds to pre- and postsynaptic α1 adrenoceptors. Activation of presynaptic α1 adrenoceptors facilitates glutamate release. Glutamate activates postsynaptic mGluR5 and induces persistent neuronal firing. Postsynaptic α2 adrenoceptors and HCN channels colocalize on dendritic spines [44]. Activation of postsynaptic α2 adrenoceptors by NE inhibits cAMP signaling and blocks HCN channels thereby increasing membrane input resistance, cell excitability and the efficacy of synaptic transmission. Cholinergic inputs induce persistent neuronal firing driven by M1 muscarinic receptors via TRPC channel-dependent mechanisms [24]. α2 adrenoceptor-mediated inhibition of HCN channels underlies NE-mediated modulation of cholinergic persistent firing. Ach: acetylcholine; Glu: glutamate.

In conclusion, we observed that NE, by acting on pre- and postsynaptic α1 adrenoceptors as well as postsynaptic α2 adrenoceptors (Figure 9), evokes robust persistent activity in pyramidal neurons in superficial layers of the PFC where the microcircuits responsible for working memory reside [3], [10]. This synergistic combination of intrinsic and network properties provides a novel mechanism for noradrenergic induction and enhancement of persistent firing in prefrontal pyramidal neurons in normal or hyperexcid states.
